# Case Report: A First Case of Spontaneous Coronary Artery Dissection Potentially Associated With Scuba Diving

**DOI:** 10.3389/fcvm.2022.855449

**Published:** 2022-04-14

**Authors:** Thabo Mahendiran, Benoît Desgraz, Panagiotis Antiochos, Vladimir Rubimbura

**Affiliations:** ^1^Department of Cardiology, Lausanne University Hospital, Lausanne, Switzerland; ^2^Underwater and Hyperbaric Medicine, Lausanne University Hospital, Lausanne, Switzerland; ^3^Yverdon-les-Bains Hospital, Yverdon-les-Bains, Switzerland; ^4^Morges Hospital, Morges, Switzerland

**Keywords:** case report, acute myocardial infarction, spontaneous coronary artery dissection, scuba diving, multimodal imaging

## Abstract

**Background:**

Scuba diving has rarely been associated with spontaneous arterial dissection. However, all documented cases have involved the cervicocranial arteries.

**Case summary:**

We report the first case of spontaneous coronary artery dissection (SCAD) potentially associated with scuba diving in a 65-year-old female with no medical history or known cardiovascular risk factors. She presented with sudden-onset chest pain during her descent whilst scuba diving on holiday. An initial ECG revealed transient abnormalities, but due to normal initial blood tests, a reassuring echocardiogram, and the resolution of her symptoms, she was discharged from hospital without a clear diagnosis. During her subsequent presentation to our hospital 1 week later, electrocardiographic evidence of an inferior myocardial infarction (MI) was noted, with an echocardiogram revealing regional wall motion abnormalities of the left ventricular inferior wall. Coronary angiography revealed the presence of a SCAD of the posterior left ventricular artery, with cardiac magnetic resonance imaging confirming the presence of an inferior MI. As recommended in the majority of cases of SCAD, this case was managed conservatively with a favorable clinical course.

**Conclusion:**

This is the first reported case of SCAD potentially associated with scuba diving. It highlights the importance of considering SCAD in patients presenting with sudden-onset chest pain during physical activity, especially in female patients (including older patients) with no cardiovascular risk factors. Furthermore, it serves as a reminder that symptoms during scuba diving are not always related to decompression illness.

## Introduction

Spontaneous coronary artery dissection (SCAD) is defined as a spontaneous, non-traumatic, non-iatrogenic, and non-atherosclerotic separation of the coronary arterial wall by intramural haemorrhage (1). It is caused by either an intimal tear or spontaneous haemorrhage from the vasa vasorum. The result is the creation of a false lumen with intramural haematoma (IMH) that can compress the true arterial lumen, leading to myocardial ischaemia and even infarction. SCAD represents an increasingly recognised cause of acute coronary syndrome (ACS), particularly among young-to-middle-aged women without cardiovascular risk factors (1). In one series of women aged <50 years old with myocardial infarction (MI), SCAD accounted for 24% of cases (2).

Scuba diving has rarely been associated with spontaneous arterial dissection, with all cases reported affecting the cervicocranial arteries (3–7). The underlying mechanism in these cases remains unclear. To date, there have been no reported cases of SCAD linked to scuba diving.

We report a first such case in a 65-year-old female scuba diver with no medical history.

## Case Presentation

A 65-year-old female with no medical history and no known cardiovascular risk factors presented with sudden onset chest pain whilst scuba diving abroad. She was a seasoned scuba diver with numerous years of experience.

The dive in question was her ninth in 5 days. She had just begun her descent with an air-only gas tank when, upon reaching a depth of 8 metres, she experienced sudden onset, central, oppressive chest pain. Despite this, she continued her descent to the target depth of 30 metres. However, due to the persistence of chest pain, she abandoned the dive and performed a controlled ascent as per recommendations. The total dive time was 30 min.

**Figure 1:**
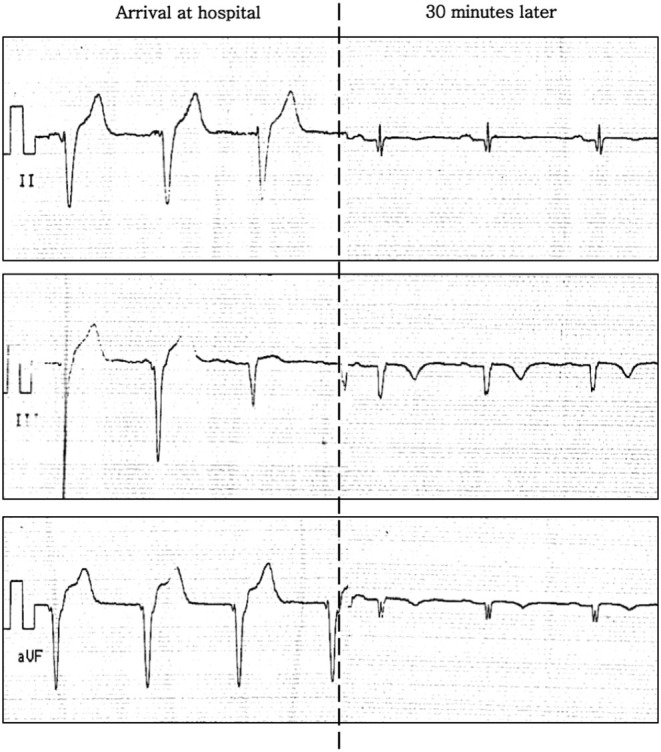
ECG (inferior leads) on presentation to hospital whilst abroad. Left: arrival at hospital. Right: 30 min later.

She presented to the local hospital ~90 min later where an initial ECG demonstrated a rhythm at ~70 beats per minutes with slightly widened QRS complexes and an absence of regular atrial activity, likely compatible with an accelerated idioventricular rhythm (Figure 1, left panel). These modifications subsequently resolved 30 min later, with a return to sinus rhythm, but with fragmented QRS complexes, Q waves, and T-wave inversion now noted in inferior leads (Figure 1, right panel). A blood test revealed normal levels of troponin and CK-MB—these tests were not repeated. An echocardiogram revealed a normal left ventricular ejection fraction (LVEF) with no regional wall motion abnormalities. The chest pain resolved ~2 h after onset, and the patient was discharged with aspirin and bisoprolol but no clear diagnosis.

**Figure 2:**
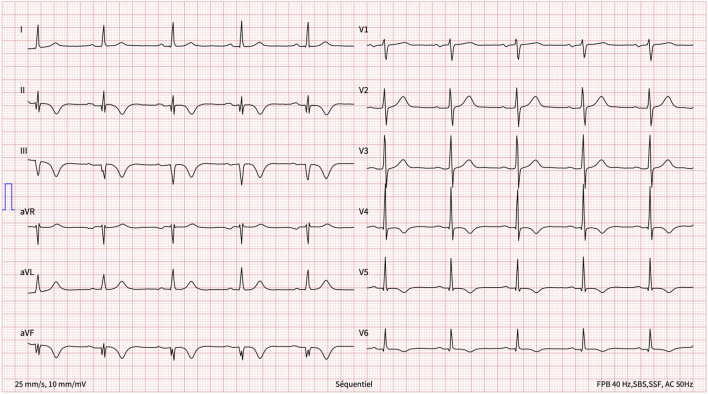
12-lead ECG on presentation to our hospital 1 week after the initial chest pain.

One week later, having returned home, she presented with new-onset, central, respiration-dependant chest pain, distinct in character from the previous chest pain. The patient was haemodynamically stable with a normal clinical examination. A 12-lead ECG revealed inferior Q waves with marked infero-lateral T-wave inversion (Figure 2).

Blood tests revealed a high-sensitivity troponin T level of 78 ng/l (normal <14 ng/l), with repeated testing over the subsequent hours/days revealing a decreasing trend that reached a nadir of 33 ng/l. Of note, C-reactive protein was slightly raised at 14 ng/l (normal <10 ng/l), but leukocytes were in the normal range.

Transthoracic echocardiography revealed an LVEF of 63% but with akinesia of the left ventricular mid-inferior wall and hypokinesia of the distal inferior wall (Supplementary Video 1).

Given the history and these findings, a subacute myocardial infarction (MI) was suspected with possible peri-infarction pericarditis. At this stage, the possible mechanisms of the MI included: a conventional type 1 MI (i.e., atherosclerotic plaque rupture) or a type 2 MI secondary to SCAD or coronary spasm, given the context of physical stress. Other differential diagnoses that were considered included myocarditis and coronary arterial gas embolism. However, the former was considered unlikely given the context, the acute onset of chest pain, and the absence of recent upper respiratory infection or gastroenteritis. The latter is phenomenon seen in decompression sickness and thus was deemed implausible given the onset of chest pain during decent, the profile of the dive, and the controlled nature of the subsequent ascent.

The patient was started on dual antiplatelet therapy (DAPT) (aspirin and clopidogrel) pending a coronary angiogram, with high-dose aspirin (500 mg tds) prescribed to manage the pericarditic chest pain. A proton pump inhibitor was also started for gastric protection.

**Figure 3:**
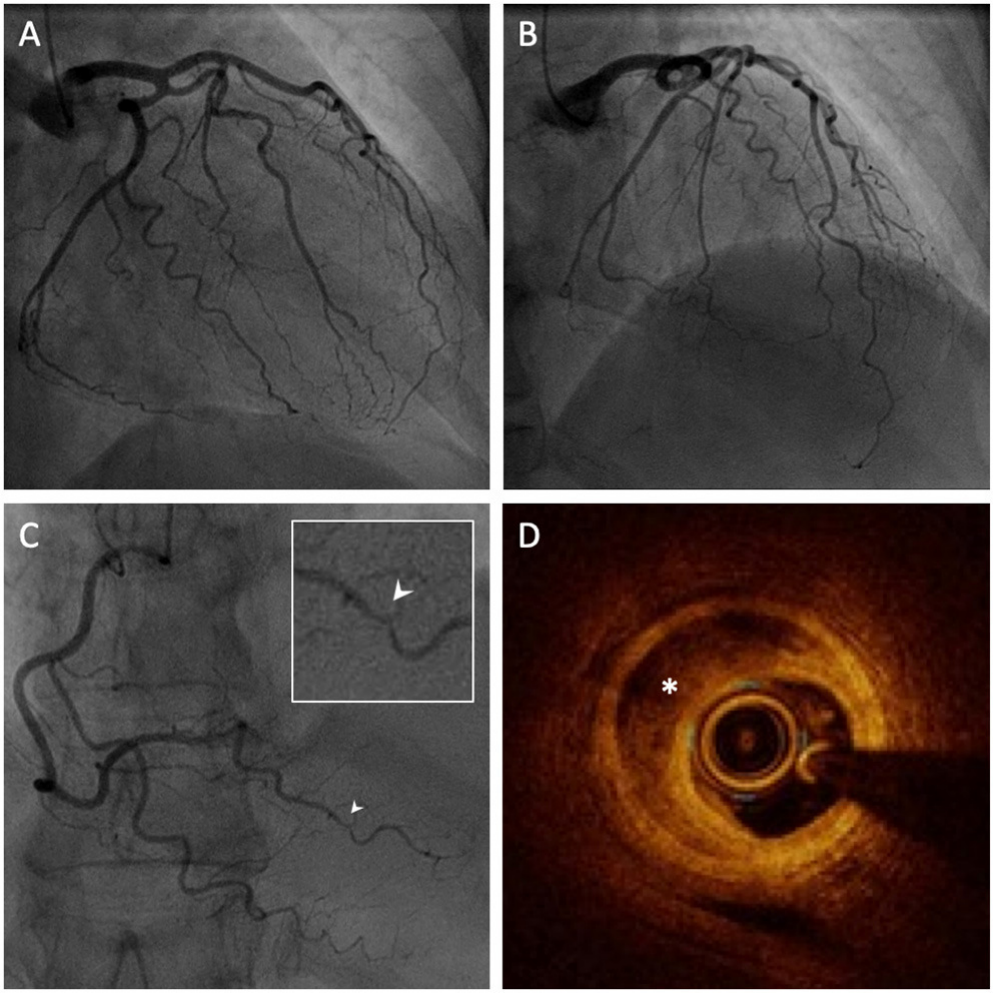
Invasive coronary angiography (ICA) with intravascular imaging by optical coherence tomography (OCT). **(A,B)** ICA of the left coronary arteries revealing an absence of significant coronary artery disease. **(C)** ICA of the right coronary artery revealing a focal irregularity of the posterior left ventricular artery (PLV), magnified in the inset (white arrow). **(D)** OCT of the PLV reveals a false lumen with intramural haematoma (white asterisk) confirming the diagnosis of SCAD.

Invasive coronary angiography revealed coronary arteries free of significant stenoses except for a subtle irregularity of the posterior left ventricular artery (Figures 3A–C). Given the suspicion of SCAD, optical coherence tomography (OCT) was performed, revealing an intramural haematoma of the posterior left ventricular artery with a patent true lumen, thus confirming the diagnosis of SCAD (Figure 3D).

**Figure 4:**
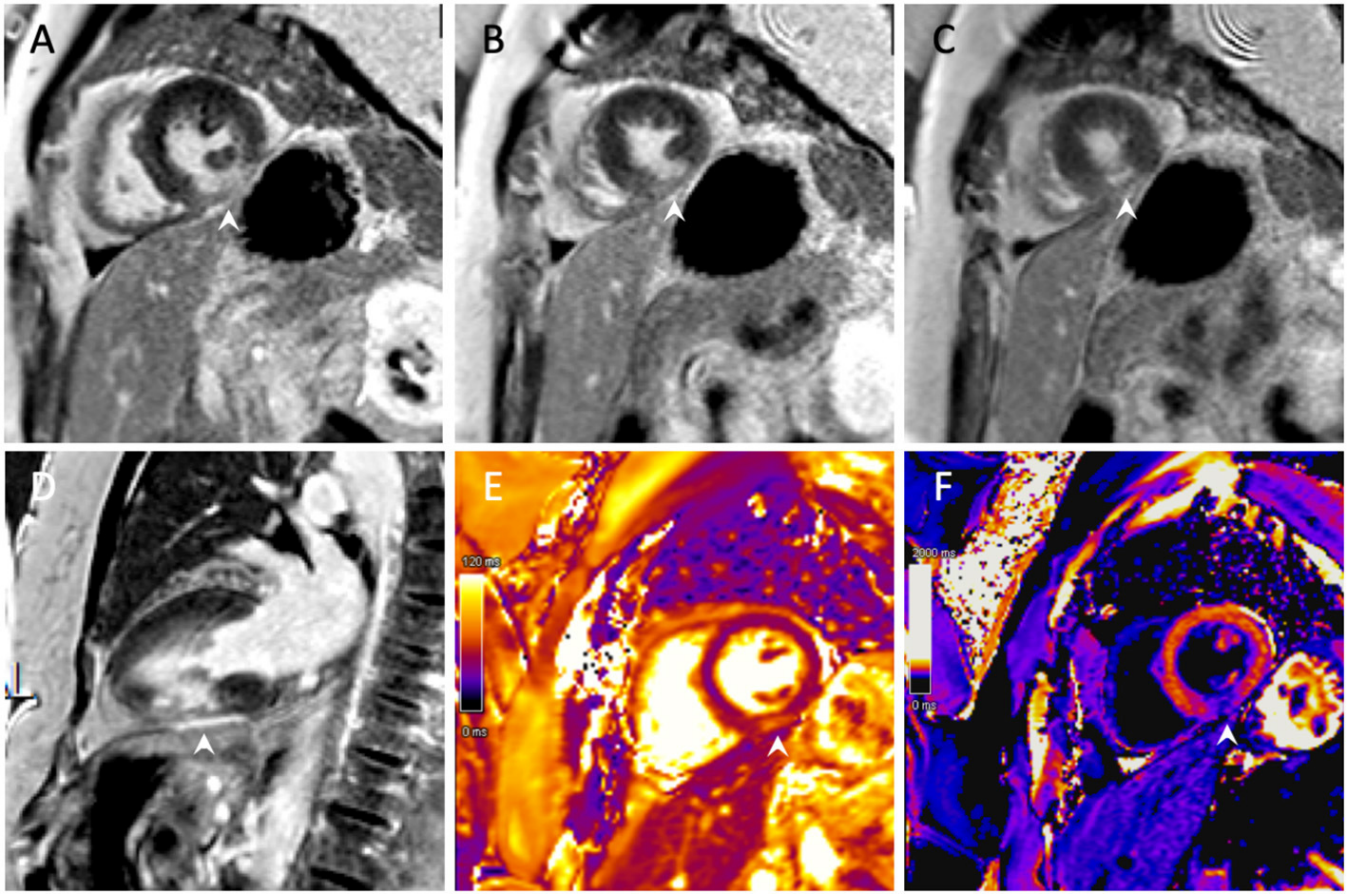
Cardiac magnetic resonance imaging with gadolinium. **(A–C)** Short-axis view with transmural late gadolinium enhancement of the mid-inferior left ventricular wall with subendocardial extension toward the apex. **(D)** Two-chamber view demonstrating an inferior infarction of the mid segment with extension toward the apex. **(E)** T2 imaging with oedema of the mid-inferior segment. **(F)** Post-contrast T1 mapping of the infarct.

Subsequent cardiac MRI (CMR) confirmed the presence of an MI of the mid-inferior wall [transmural late gadolinium enhancement (LGE)] with extension to the distal inferior territory (subendocardial LGE) (Figure 4). Additionally, LGE of the pericardium in contact with the zone of infarction was also noted, suggesting a localised pericarditis.

As per current recommendations (8), given the absence of haemodynamic instability, and the presence of normal flow in the culprit coronary artery, the SCAD was managed conservatively without revascularisation. To treat the pericarditis, high-dose aspirin was continued for a total of 2 weeks along with a 3-month course of colchicine as per current guidelines (9). Low-dose aspirin (100 mg od) was subsequently continued long-term. Clopidogrel was discontinued due to the lack of clear evidence for the use of dual antiplatelet therapy in this context. Due to the presence of a normal LVEF, ACE inhibitors and beta blockers were not prescribed.

The patient's symptoms resolved during the admission and she was discharged from hospital 6 days later. The follow-up period was unremarkable with an absence of chest pain recurrence. Of note, routine screening for fibromuscular dysplasia by duplex ultrasonography was negative. The screen was completed with computed tomography angiography in ambulatory care.

## Discussion

To the best of our knowledge, this is the first reported case of SCAD potentially associated with scuba diving. The case was even more unusual due to the age of patient, given that SCAD is more typically associated with women <50 years old.

The transient ST-elevation, inferior Q-waves, and transmural, inferior infarct suggest that our patient had suffered an inferior ST-elevation myocardial infarction (STEMI). Of note, STEMI is the clinical presentation in up to 30% of cases of SCAD (10). Numerous factors likely played a role in the missed diagnosis when the patient first presented to hospital whilst abroad, including the transient ECG abnormalities, reassuring initial blood tests and echocardiogram, and the resolution of chest pain. However, another likely contributing factor is the lack of awareness of SCAD as a potential cause of acute chest pain and ACS, particularly in the context of physical stress.

SCAD has historically been considered a rare cause of MI, but contemporary angiographic data now recognises it as the underlying cause in 4% of ACS cases (11). SCAD has a strong preponderance in females (~90%), with mean age at presentation ranging from 44 to 53 years according to contemporary data (8). It is thought that this predilection is related to female sex hormones and their influence on vascular connective tissue. This likely explains why 27% of infarctions during pregnancy and 50% of those post-partum are due to SCAD (8).

However, despite the clear predisposition for young women, SCAD can occur in older patients, as highlighted by our case. In a large Canadian series, Saw et al. found that 55% of SCAD cases were in post-menopausal women, with ~10% of patients being 65 years old or more (10). These data suggest that SCAD does occur in older patients, although such patients represent a significant minority.

Our understanding of the pathophysiology of SCAD has advanced considerably over recent years. Conceptually, the drivers of the condition can be divided into: (1) predisposing conditions and (2) precipitating stressors. The recent Canadian series found that the most common predisposing condition was fibromuscular dysplasia (FMD) (31.1%), followed by systemic inflammatory diseases (4.7%), the peripartum period (4.5%), and connective tissue disorders (3.6%) (8). However, ~50% of cases were deemed idiopathic with no identified predisposing condition (10).

As for potential precipitating stressors, triggers include intense physical exercise, intense emotional stress, Valsalva-type activities (e.g., retching, vomiting, bowel movement, coughing), and recreational drug use (e.g., cocaine, amphetamines, methamphetamines) (1). This was confirmed in the series by Saw et al. which identified precipitating stressors in the majority of cases of SCAD, with emotional stress reported in 50.3% of cases, and physical stress in 28.9% (9.8% lifting >50 pounds) (10).

It is likely that the physical stress associated with scuba diving was a trigger of SCAD in our case. It is thought that intense physical activity can provoke a Valsalva-like increase in intrathoracoabdominal pressure which can be transmitted to coronary arteries as shear stress. A likely contributory mechanism is a surge in catecholamines that leads to increased myocardial contractility or vasospasm, a subsequent increase in arterial shear stress, and ultimately intimal rupture or disruption of the vasa vasorum. This latter mechanism is also the likely mechanism for SCAD cases linked to intense emotional stress (1). In addition, diving exposes the cardiovascular system to specific stressors such as hydrostatic pressure, hyperoxia-induced vasoconstriction, and elevated cardiac filling pressures. Cold and exercise can further amplify these effects, as well as increasing sympathetic nervous system activity (12, 13). Finally, vigorous ear pressure equilibration via the Valsalva maneuver could have served as an additional contributing stressor in the development of SCAD in our case. Importantly, the occurrence of symptoms at the beginning of the dive, during descent, make decompression illness an unlikely contributing factor.

Invasive coronary angiography (ICA) represents the first-line investigation in suspected cases of ACS. However, in the case of SCAD it has important limitations as it does not image the arterial wall. An ICA-based classification system has been developed that divides SCAD into three types (14). Type 1 describes the pathognomonic appearance of arterial wall contrast staining with multiple radiolucent lumens. Type 2 describes the presence of a long, diffuse narrowing which may be bordered by normal artery segments proximally and distally (type 2A), or extend to the end of the vessel (type 2B). Finally, type 3 refers to a focal stenosis that mimics atherosclerosis (as seen in the present case), thus requiring intracoronary imaging to confirm diagnosis. Options for intracoronary imaging include OCT or intravascular ultrasound (IVUS). Although IVUS offers higher penetration permitting better visualisation of the depth of IMH, OCT is preferred overall due to its superior spatial resolution (10–20 μm vs. 150 μm) allowing for better visualisation of intimal tears, intra-luminal thrombi, false lumens and IMH (1).

As for the management of SCAD, current recommendations are based upon expert consensus and observational data due to the absence of randomised trials that address the subject. The vast majority of cases can be managed conservatively, an approach that has been confirmed by the angiographic resolution of SCAD lesions commonly seen after conservative management (15). However, in patients presenting with high-risk features - namely symptoms of ongoing ischaemia, haemodynamic compromise, or left main dissection - revascularisation with percutaneous coronary intervention (PCI) or coronary artery bypass grafting is recommended (14). However, it should be noted that PCI is associated with high rates of technical failure (up to 50%), which relates in part to the fragility of the vessel wall and the risk of propagation of the dissection or occlusion of side branches (16, 17). Of note, the use of a cutting balloon to decompress the IMH into the true lumen may reduce to risk of SCAD propagation if stenting is subsequently employed (18).

Recommendations for medical therapy in SCAD are limited by a lack of randomised controlled trials comparing different pharmacological treatment strategies. In patients who undergo stenting, DAPT for 12 months followed by long-term aspirin therapy is recommended as per current ACS guidelines (19, 20). The benefit of DAPT in the absence of coronary stenting is unclear, especially since previous studies have shown that the principal cause for reduced coronary flow is not thrombus, but compression by the IMH in the false lumen (1). As a result, in patients managed conservatively, long-term aspirin appears reasonable, in particular given its favorable side effect profile, while the addition of clopidogrel for 1–12 months post-SCAD remains debatable (8). Of note, there may be a role for repeat coronary imaging during follow-up to assess for SCAD-healing and facilitate decision-making regarding the duration of antithrombotic therapy. Additional potential medical treatment includes beta blocker therapy which is thought to reduce arterial shear stress, and has been associated with reduced SCAD recurrence in one observational study (21). However, in general, statins are reserved for patients with conventional indications for treatment independent of their SCAD event.

As for the management of SCAD post-discharge, cardiac exercise rehabilitation has been shown to be safe and beneficial (8). Longer-term, patients should be counseled against isometric or extreme exercise given the clear-cut reported associations. Otherwise, based on currently available data, it appears reasonable for patients to return to full activity following SCAD (1, 8). In our case, a specialist evaluation with a diving physician will be organised before considering resumption of scuba diving.

Despite the initially missed diagnosis of SCAD whilst abroad, our patient received appropriate initial medical treatment (aspirin and bisoprolol) pending her presentation to our hospital where the diagnosis of SCAD was confirmed. As seen in the majority of cases, conservative management resulted in a favorable clinical course, with a normal LVEF despite localised regional wall motion abnormalities, and coronary angiography confirming a resolving SCAD with a patent true lumen. Among patients hospitalised and diagnosed with SCAD, both short- and long-term mortality appears to be low. However, it is important to recognise that the true incidence of SCAD and its outcomes remain unknown due to potential under diagnosis and ascertainment bias. In particular, it is likely that SCAD is responsible for a non-trivial proportion of patients that present with sudden cardiac death who never make it to hospital. Among patients diagnosed with SCAD, recurrence rates of ~12% have been reported highlighting the importance of close follow-up of SCAD survivors (1).

## Conclusion

Scuba diving has rarely been associated with spontaneous arterial dissection, with all documented cases affecting the cervicocranial arteries. To the best of our knowledge, this is the first reported case of SCAD potentially associated with scuba diving. This case serves as a reminder that symptoms during scuba diving are not always related to decompression illness. Furthermore, it highlights the importance of considering SCAD in patients presenting with sudden-onset chest pain, in particular in female patients with no cardiovascular risk factors. Finally, this case underscores that, contrary to widespread believe, SCAD can occur in older patients and thus should be considered as a cause of ACS in such patients.

## Data Availability Statement

The raw data supporting the conclusions of this article will be made available by the authors, without undue reservation.

## Ethics Statement

Written informed consent was obtained from the individual(s) for the publication of any potentially identifiable images or data included in this article.

## Author Contributions

TM drafted the manuscript and performed the echocardiogram. BD, VR, and PA provided critical review of the manuscript. PA performed the cardiac MR. VR performed the coronary angiography. BD was responsible for patient follow-up. All authors contributed to the management of this case, contributed to the article, and approved the submitted version.

## Funding

Open access funding provided by University of Lausanne.

## Conflict of Interest

The authors declare that the research was conducted in the absence of any commercial or financial relationships that could be construed as a potential conflict of interest.

## Publisher's Note

All claims expressed in this article are solely those of the authors and do not necessarily represent those of their affiliated organizations, or those of the publisher, the editors and the reviewers. Any product that may be evaluated in this article, or claim that may be made by its manufacturer, is not guaranteed or endorsed by the publisher.

## Supplementary Material

Supplementary Video 1 Transthoracic echocardiography (apical two-chamber view) demonstrating regional wall motion abnormalities of the inferior wall of the left ventricle.
